# SEMA5A-PLXNB3 Axis Promotes PDAC Liver Metastasis Outgrowth through Enhancing the Warburg Effect

**DOI:** 10.1155/2023/3274467

**Published:** 2023-01-27

**Authors:** Kun Wang, Min He, Xin Fan, Jian Zhou, Jian Yang, Lin Wang, Zhihong Zhao, Chun Dai, Zixiang Zhang

**Affiliations:** ^1^Department of General Surgery, The First Affiliated Hospital of Soochow University, Suzhou, 215006 Jiangsu Province, China; ^2^Department of General Surgery, Affiliated Hospital of Jiangsu University, Zhenjiang, 212001 Jiangsu Province, China; ^3^Department of Biliary-Pancreatic Surgery, Ren Ji Hospital, School of Medicine, Shanghai Jiao Tong University, Shanghai, China; ^4^Department of General Surgery, People's Hospital of Yangzhong, Yangzhong, 212299 Jiangsu Province, China

## Abstract

Patients bearing liver metastasis of pancreatic adeno carcinoma (PDAC) suffer from poor prognosis due to its short duration and high mortality. Complex tumor microenvironment (TME) exists in liver metastatic niches, and tumor-associated macrophages (TAMs) have play vital roles in metastasis generation and outgrowth. We have discovered that M2 type TAM-derived SEMA5A could bind to its tumor cell-expressed receptor PLXNB3 to promote tumor cell proliferation and outgrowth. We utilized liver metastasis samples of PDAC patients, intrasplenic injection mouse models, and *Kras*^G12D^/*Trp53*^R172H^/*Pdx1*-Cre (KPC) mouse models for *in vivo* study. In mechanism investigation, we have discovered that SEMA5A-PLXNB3 axis could achieve tumor cell proliferation and survival via enhancing aerobic glycolysis termed as the Warburg effects. Targeting this axis may be a potential therapeutic approach for PDAC patients with unresectable liver metastasis.

## 1. Introduction

Pancreatic adenocarcinoma (PDAC) has been one of the malignancies with poor prognosis; its high fatality rate and metastasis behavior lead to a 5-year survival rate of less than 6% [1, 2]. The most common target organ for PDAC distant metastasis is the liver, and patients bearing liver metastasis could not get access to surgery and suffer from frequently relapsing. Thus, it is important to study the mechanism and potential therapeutic targets for liver metastasis of PDAC.

Tumor microenvironment (TME) consists of complex members including cancer-associated fibroblasts (CAFs) [3, 4], tumor-associated macrophages (TAMs) [5–7], natural killer cells (NK cells) [8], and regulatory T cells (T-regs) [9–11], which share reciprocal functions to each other. Lots of study have been done to emphasize the importance of TME, while the relationship of TAMs and liver metastatic niches of PDAC remains largely unknown. TAMs usually aggregated around tumor niches into enriched extracellular matrix (ECM) or even deeply infiltrated into the intervals of niches to exert their promotive or antieffects on tumor cells [12, 13]. Accumulated evidence has demonstrated that M2 type macrophages could stimulate proliferation, migration, and invasion activity of tumor cells mainly via secreting various chemokines or other proteins [5, 6, 12, 14]. Tumor growth promotive protein from M2 TAMs could be detected at high concentration to immerse tumor cells for niche filtration and expansion.

Axon guidance family is vital in embryonic development and neuron growth, which can act as a leader to decide and induce the directions of axons of neurons [15]. While in recent years, more and more reports have discovered that this family has played critical roles in tumorigenesis and metastasis [16, 17]. Among them, semaphorin is one of the most important families mentioned in tumor-related pathways [17, 18]. Semaphorin is mainly recognized responsible for extracellular signaling transduction and also essential for cell development and even tumor growth [19, 20]. Diverse structures of SEMA family members are detected so far, and their cellular locations differ from each other. It has been discovered that SEMA2 and 3 family members were all secreted proteins; while though SEMA5 family members were usually considered as transmembrane proteins, several studies have demonstrated that they would be cleaved and released into cellular matrix [21]. The canonical receptors of SEMAs were PLXNs, which were expressed on the membrane of cells and received the signals from SEMAs before triggering the downstream pathway.

In this study, we set off from evaluating the infiltration of M2 TAMs in patients derived liver metastasis tissues and discovered the enrichment of extracellular SEMA5A in M2 TAM aggregation area. We also found that PLXNB3, one of the canonical receptors of SEMA5A, was also aberrantly overexpressed on M2 TAM-infiltrated metastatic niche resident tumor cell membranes. We established intrasplenic injection mouse model to mimic the physiological progress of PDAC liver metastasis and assessed by CT combined in vivo imaging with organ reconstruction. We also generated *Kras*^G12D^/*Trp53*^R172H^/*Pdx1*-Cre (KPC) mouse models which could form liver metastasis of PDAC spontaneously. In mechanism exploration, we discovered that SEMA5A-PLXNB3 axis efficiently facilitated the Warburg effects via upregulating cell stress-related genes and glycolysis function involved key enzymes, thus promoting tumor cells growth.

Our study has put forward a novel insight into liver metastasis of PDAC and might be a promising target for metastatic PDAC.

## 2. Material and Methods

### 2.1. Constructs and Reagents

Antibodies of CD163, IBA1, MHC-II, CD11c, SEMA5A, CK19, PLXNB3, PLXNA3, PLXNA3, PKM2, PGK1, and Ki67 were purchased from Abcam, UK. Anti-rabbit Alexa Fluor 488, anti-rabbit Alexa Fluor 596, anti-mouse Alexa Fluor 488, anti-mouse Alexa Fluor 596, and HRP-conjugated secondary antibodies were purchased from Jackson, USA. Cell Counting Kit-8 (CCK-8) was purchased from Dojindo Molecular Technologies, JP. G-Dynabeads was purchased from Life Technologies, USA. In Situ Cell Death Fluorescein Kit TUNEL and SYBR Premix Ex Taq were purchased from Roche, CH. Glucose assay kit, oligomycin, and 2-deoxyglucose were purchased from Sigma-Aldrich, USA. Lactate Assay Kit was purchased from BioVision, USA. DAB staining kit was purchased from Cell Signaling Technologies, GER. Episomal expression vector with pCEP-Pu-Strep II-tag, vector used for carrying shRNA, and vector for PLXNB3 overexpression were all purchased from Gene Pharma Technologies, PRC. DAPI staining reagent was purchased from Beyotime Technologies, PRC.

### 2.2. Clinical Samples

Clinical samples involved in this study were all from Renji Hospital, Shanghai Jiaotong University School of Medicine. We claimed that all samples used in our study (the sample collections, tissue preparations, and experiments performance) were approved by local ethics committee; the approval number is RA-2019-116.

All these patients were diagnosed by both clinical surgeons and professional pathologists, and they were informed to participate in the study and gave their written consent to participate for this research.

Our study on liver metastasis contains 20 cases of patients with liver metastasis of PDAC. The sample of liver metastasis was collected for IHC-P staining, IF staining, or real-time PCR.

### 2.3. Animals

Male C57BL/6J mice (6-8 weeks) and KPC mice (about 3-4 months) used in this study were all received humane care, housed, and fed in accordance with the Guide for the Care and Use of Laboratory Animals prepared by the National Academy of Sciences and published by the National Institutes of Health. Our animal experiments were approved and invested under approved protocol number 20141204 assigned by the Research Ethics Committee of East China Normal University.

In this research, we used intrasplenic injection model to study the liver metastasis of PDAC. Here, we used murine cell line Kpc1199, which was derived from *Kras*^G12D^/*Trp53*^R172H^/*Pdx1*-Cre (KPC) mouse model. For injection preparation, we first stably transfected plasmid carrying GFP/luciferase into Kpc1199 followed by flow cytometry sorting. Then, the gained Kpc1199 cells were transfected shCTRL or sh*PLXNB3* to perform injections. These two group cells were suspended in 20 *μ*l medium without FBS at a concentration of 2 × 10^6^ cells per mouse before injection. We anesthetized C57BL/6J mice via 2.5% isoflurane inhalation. Then, mice were exerted surgery to expose the spleen, and tumor cells mentioned above were injected at a speed of 2 *μ*l/s. The needle would be left in the spleen for at least 30 s to prevent leakage before wound closure.

For KPC mice, this model is a spontaneous PDAC mouse model which could generate PDAC and liver metastasis at about 3-4 months old. To gain the liver containing metastatic niches, we observed the living status and some specific characters (e.g., the hardening of pancreatic and hepatic region) of each mouse every day since 3 months old and harvest the samples for further research.

### 2.4. Immunohistochemistry and Immunofluorescence

Human liver metastasis samples or mouse model liver metastasis samples were collected and performed paraformaldehyde fixation. At the beginning, xylene in gradient alcohol at different concentration (from 100% to 75%) was used to perform deparaffinization and rehydration. Citrate buffer at 100°C was used for antigen retrieval which lasted 20 min. Then, endogenous peroxidase inactivation using exposure of 0.3% hydrogen peroxide in methanol to slides was performed. After PBS washing, bovine serum albumin (BSA) was used to blocking for 1.5 h before incubation of primary antibodies overnight. For IHC-P staining, HRP-conjugated secondary antibody exposure was performed 2 h at room temperature. After washing 3 times, DAB staining was performed about 10 s-30 s according to target protein enrichment. Hematoxylin staining was then performed on all slides followed by 10 min washing. For IF staining, the IF secondary antibodies were performed and nuclei was stained with DAPI. NIKON Eclipse ci (JP) equipped with NIS_F_Ver43000_64bit_E software was used for observation and snapping. For immunohistochemistry snapping, the magnification is 200x, and for immunofluorescence, the magnification is 400x.

### 2.5. Cell Culture

In this study, human PDAC cell lines PANC1, BxPC3, AsPC1, and CAPAN1, human normal pancreatic cells HPNE, and murine PDAC cell lines Panc02 and KPC1199 were all purchased from Cell Bank of Chinese Academy of Sciences (China). All these cell lines were culture and maintained according to official instructions. The complete medium consists of DMEM medium, 10% fetal bovine serum (FBS), and 1% antibiotic mixture. All cells were kept at 37°C with 5% CO_2._

### 2.6. SEMA5A and SEMA5A-*Δ*TSP Protein Expression and Purification

We utilized episomal expression vector with pCEP-Pu-Strep II-tag carrying SEMA5A or SEMA5A-*Δ*TSP. 293-T cells were then stably transfected with these reconstructed plasmids which could then secreted recombined SEMA5A (rSEMA5A) and recombined SEMA5A-*Δ*TSP (rSEMA5A-*Δ*TSP). Then both groups were filtered by cultured in medium containing puromycin at 5 *μ*g/ml and maintained at 2 *μ*g/ml. After validation of secretions, we collected the cell supernatants of these two groups of tumor cells and added protease inhibitors for further use. The collected cell supernatants were carefully applied for Strep Tactin sepharose column followed by washing of bind buffer. At last, elution buffer containing 2.5 mM desthiobiotin was used for harvesting SEMA5A or SEMA5A-*Δ*TSP. The concentrations of these two proteins were determined by NanoDrop 2000.

### 2.7. Cell Transduction

The lentivirus carrying shRNA targeting PLXNB3 was performed (5′-AGCAGATGGTGGAGAGGT A-3′). 1x HitranasG transfection reagent was utilized for transfection. Then, the transfected tumor cells were filtered by puromycin at a concentration of 5 *μ*g/ml fist and maintained at 2 *μ*g/ml.

### 2.8. Cell Viability Assay

Cell counting kit-8 (CCK-8) was performed to evaluate the tumor cell viability. Robust PANC1 at 5000 cells/100 *μ*l, BxPC3 at 6000 cells/100 *μ*l, and AsPC1 at 4000 cells/100 *μ*l were planted into 96 well plates. These cells were then allowed to grow for adaption for 1 day and then were used to perform CCK-8 assay. For experiment, the CCK-8 reagent was diluted by complete medium at a ratio of 1 : 10 before incubation for 1 h. Then, microplate reader was used to measure the incubated holes at 450 nm. The CCK-8 reagents were then rapidly removed after measurement and cells were continued to be cultured in complete medium. Every group containing 5 duplicated holes for repeat.

### 2.9. Real-Time PCR

RNA extraction was performed before cDNAs were transcribed using PrimeScript RT-PCR kit. SYBR Green Ex Taq was used for 7500 real-time PCR system (Applied Biosystems, USA). Sequences of primers are as shown in Table 1

### 2.10. Seahorse Assay

The ECAR measurement was performed on Seahorse XF96 Flux Analyser (Seahorse Bioscience). PANC1 at 15000 cells and BxPC3 at 20000 cells were seeded on XF-96-wll plate. Then, these cells were deprived from FBS for starvation for 24 h. The real-time of ECAR is recognized as an indicator of the net proton loss during glycolysis. At first, all groups of tumor cells in plates were incubated with unbuffered medium. Then a series of treatments were performed. In the follow-up, these cells were exposed in subsequence to glucose (10 mM), oligomycin (1 mM), and 2-deoxyglucose (80 mM). The related data would be recorded. Among these, oligomycin is used to prevent proton flow via mediating the ATP synthase and induce the maximal glycolytic metabolism. The ECAR results displayed in this research were normalized to total protein content and reported as mpH/min.

### 2.11. Intrasplenic Mouse Model and CT Combined In Vivo Imaging with Organ Reconstruction

For in vivo imaging, mice bearing liver metastasis were intraperitoneally injected 50 mg D-luciferin (200 *μ*l) before being anesthetized with 2.5% vaporized inhaled isoflurane. We then waited for 2 or 3 min for D-luciferin reaction for optimal observation. In Vivo Imaging System (IVIS) Spectrum (Caliper Life Sciences, Waltham, MA) was used for detecting metastatic niches. The CT image and organ reconstruction were generated and merged by AI of this system. The values of CT combined in vivo imaging with organ reconstruction were gained by AI system to calculate the average firefly signals in one preset cube.

### 2.12. Ex Vivo Assay

For ex vivo assay, fresh metastasized liver of KPC mice was harvested before experiment. The liver containing metastatic niches was sliced in half on ice to ensure that the histological characteristics of left part (L) and right part (R) were same. Then, L part was placed in preheated complete medium with administration of rSEMA5A (30 nM), while R part was place in medium with administration of rSEMA5A*Δ*TSP (30 nM). The duration is 2 hours. Then, tissues were collected for further analysis.

For detection, the obtained tissues (L and R) were performed section on the slice side before IF staining. The expression and location of HIF1*α* or the coexpression of PKM2 or PGK1 with proliferation marker KI67 was thus performed.

### 2.13. Glucose Uptake and Lactate Production Measurement

All cells involved in this test were planted in 6-well plate and performed 24 h starvation before the tests. Then, glucose assay kit and lactate assay kit were performed. All experiments were performed according to manufacturer's instructions and related researches [22, 23]. In brief, culture medium of these cells was first clarified by centrifugation followed by filtration through filters (0.22 *μ*m) to prepare for glucose uptake and lactate production measurement. For glucose uptake, this value is recognized by the net content of the original glucose concentration deduced from the measured glucose concentration in the medium. For lactate production, it is directly measure by a kit.

### 2.14. Data Analysis

The IHC-P and IF staining were performed on the tissues of PDAC patients: *n* = 20 patients, 3 fields assessed per sample; real-time PCR measured expressions of PLXN family in TAMs slightly or severely infiltrated metastatic niches of PDAC patients: *n* = 5 samples per group; the Kaplan-Meier analysis on PDAC patients bearing dual high expressions of SEMA5A and PLXNB3 or single high expression of SEMA5A in liver metastatic niches: *n* = 8 patients per group; relationship of PLXNB3, PLXNA3, or PLXNA4 and CD163 (E) or SEMA5A: *n* = 30 samples; for mouse experiments: *n* = 6 mice per group; for CCK-8 results:5 duplicated holes per group; and for glucose uptake/lactate production/enzyme expression: 3 repeats per group.

We used repeat measure ANOVA for CCK-8 results (5 duplicated holes per group); we used two tailed unpaired *t*-test for measuring difference between groups; we used chi-square test for measuring the relationship of two related gene expressions according to staining scores; we used Pearson's product-moment correlation coefficient to measure relationship of two gene expressions according to real-time PCR.

All data performed in this study were treated by SPSS 14.0 and GraphPad 7.0.

## 3. Results

### 3.1. SEMA5A Is Derived from Infiltrated M2 TAMs

**Figure 1:**
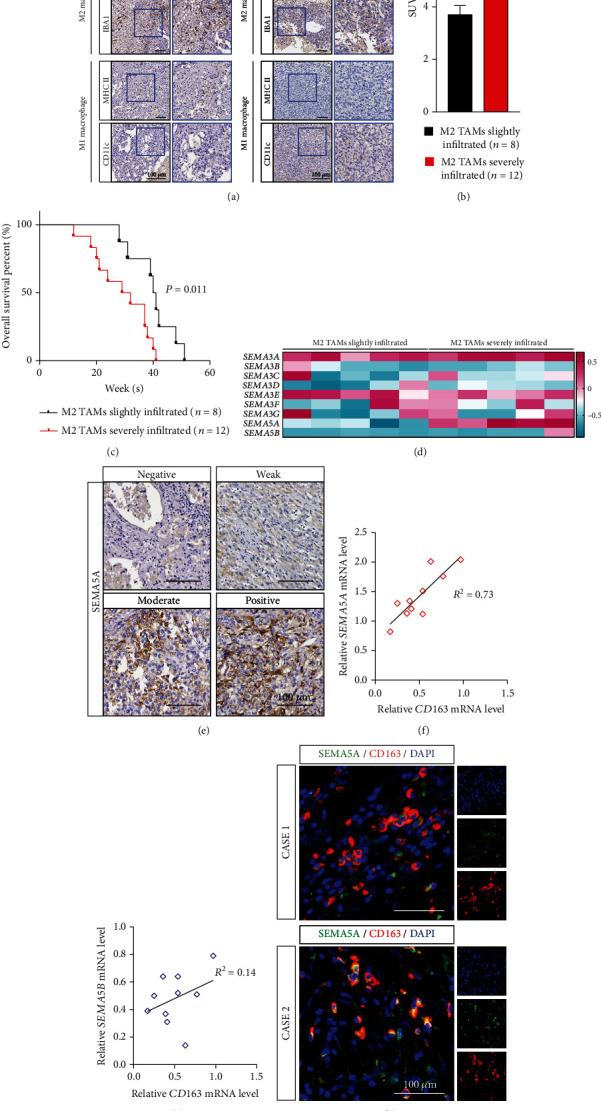
SEMA5A is secreted from M2 macrophages in PDAC liver metastatic niches. (a) Abundance and distribution of infiltrated TAMs in liver metastasis of PDAC were measured by IHC-P staining on M1 macrophage markers (CD163 and IBA1) or M2 macrophage markers (MHCII and CD11c) (*n* = 20 patients, 3 fields assessed per sample). Scale bars, 100 *μ*m. (b) The SUV difference between patients bearing TAMs slightly infiltrated metastatic niches (*n* = 8) and TAMs severely infiltrated ones (*n* = 12). Mean ± s.e.m.; two tailed unpaired *t*-test, ^∗∗^*P* < 0.01. (c) The Kaplan-Meier analysis on patients bearing TAMs slightly infiltrated metastatic niches (*n* = 8) and TAMs severely infiltrated ones (*n* = 12) was performed. (d) Real-time PCR measured expressions of secreted semaphorin family in TAMs slightly or severely infiltrated metastatic niches (*n* = 5 samples per group). (e) IHC-P staining on SEMA5A demonstrating its distribution in liver metastatic niches of PDAC (*n* = 20 patients, 3 fields assessed per sample). Scale bars, 100 *μ*m. (f, g) The relationship of CD163 expression and SEMA5A (f) or SEMA5B (g) was measured (*n* = 10 samples). (h) IF staining on SEMA5A and CD163 in PDAC liver metastatic niches displaying their colocation situations (*n* = 20 patients, 3 fields assessed per sample). SEMA5A, green; CD163, red; DAPI, blue; scale bars, 100 *μ*m.

To uncover the how TAMs affect tumor cell metabolism thus leads to poor prognosis of hepatically metastatic PDAC; we first set out to investigate the category and distribution of TAMs. IHC-P on liver metastatic niches of patients bearing PDAC have demonstrated that IBA1- or CD163-positive M2 TAMs have predominated in TAMs infiltrating niches compared to MHCII or CD11c expressed ones (Figure 1(a)). Especially, the SUV of PET-CT in M2 TAMs severely infiltrated was much higher than those with little infiltration (Figure 1(b)), which indicated relationship between cell metabolism and M2 TAM distributions. Moreover, the aggregation and infiltration of M2 TAMs could also lead to poor prognosis of metastasized PDAC (Figure 1(c)). It has been reported that secreted SEMA family has played vital roles in metastasis of several kinds of cancers and closely related to TME; we next studied the expressions of which in liver metastasis with or without M2 TAM infiltration. Interestingly, some SEMA family members including SEMA3A and SEMA3E have displayed equal high expression in both groups, indicating that the source of which was not TAMs, while SEMA5A, another important secreted ligand, has been found to upregulate in TAM-enriched metastasis tissues (Figure 1(d)). Further confirmation on liver metastasis slices was also performed; the results of which were in accordance with our formed discovery (Figure 1(e)). We also compared the relationship of CD163 and SEMA5B, another SEMA5 family member, in liver metastasis samples and gained that only SEMA5A possessed strong relationship with M2 TAMs (Figures 1(f) and 1(g)). Finally, we also performed IF staining to find that SEMA5A was derived from CD163-positive M2 TAMs (Figure 1(h)).

Taken together, we have discovered that M2 TAMs would lead to poor prognosis in PDAC patients and act as a source of SEMA5A.

### 3.2. SEMA5A Is Collocated with Its Receptor PLXNB3 Receptor in Tumor

**Figure 2:**
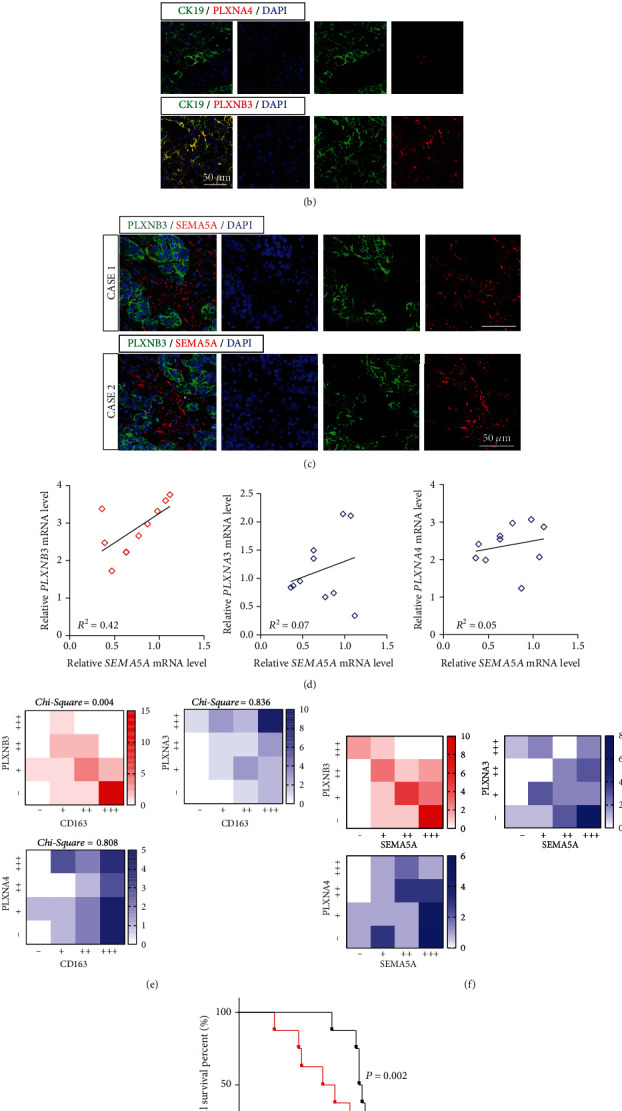
Aberrant dual high SEMA5A and PLXNB3 expressions lead to poor prognosis. (a) Real-time PCR measured expressions of PLXN family in TAMs slightly or severely infiltrated metastatic niches (*n* = 5 samples per group). (b) IF staining on CK19 and PLXNA3 (top), PLXNA4 (middle), or PLXNB3 (bottom) in liver metastatic niches demonstrating PLXNs locations (*n* = 20 patients, 3 fields assessed per sample). CK19, green; PLXNs, red; DAPI, blue; scale bars, 50 *μ*m. (c) IF staining on PLXNB3 and SEMA5A in liver metastatic niches displaying their colocation relationship (*n* = 20 patients, 3 fields assessed per sample). PLXNB3, green; SEMA5A, red; DAPI, blue; scale bars, 50 *μ*m. (d) Relationships of PLXNB3 (left), PLXNA3 (middle), or PLXNA4 (right) mRNA expressions and SEMA5A were measured (*n* = 10 samples). (e, f) Heat map showing the coexpression situations of PLXNB3, PLXNA3 or PLXNA4, and CD163 (e) or SEMA5A (f) in liver metastatic niches (*n* = 30). (g) The Kaplan-Meier analysis on PDAC patients bearing dual high expressions of SEMA5A and PLXNB3 or single high expression of SEMA5A in liver metastatic niches (*n* = 8 patients per group).

We next aimed to find out the receptor of SEMA5A in PDAC liver metastasis. It has been concluded that the potential receptors were including PLXNA3, PLXNA4, and PLXNB3, which were discovered upregulated in SEMA5A highly expressed niches in varying degrees (Figure 2(a)). We next performed IF staining on these tissues and demonstrated that it was PLXNB3 which located on CK19^+^ metastasized tumor cells in niches (Figure 2(a)). What is more, we also illustrated that SEMA5A was enriched in stoma around PLXNB3-expressed niches (Figure 2(c)). We have also calculated the relationship of SEMA5A and its three potential receptors in PDAC liver metastasis; the results have demonstrated our hypothesis (Figure 2(d)). Furthermore, we have assessed their relationships via scoring their IHC-P staining and got that PLXNB3 was coexpressed with either CD163 or SEMA5A in PDAC liver metastasis tissues (Figures 2(e) and 2(f)). At last, we have selected 16 PDAC patients whose liver metastasis niches were of high SEMA5A abundance and divided them into two groups according to PLXNB3 expressions before prognosis assessment. The results have elucidated that the dual high expression of SEMA5A and PLXNB3 would lead to poor prognosis (Figure 2(g)).

In summary, these results have shown that SEMA5A and its receptor PLXNB3 were discovered to enhance coexpression in liver metastasis of PDAC and predict poor prognosis.

### 3.3. SEMA5A-PLXNB3 Axis Promoted Liver Metastasis *In Vivo*

**Figure 3:**
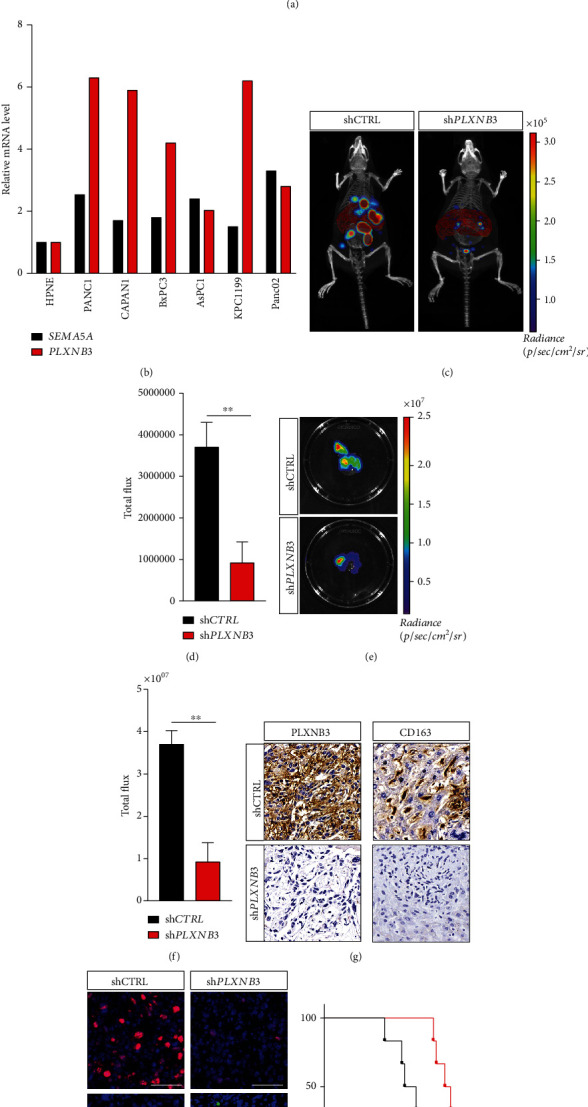
SEMA5A-PLXNB3 axis promotes liver metastasis *in vivo.* (a) The coexpressions of CK19 and PLXNB3 were performed by IF staining in spontaneous PDAC liver metastasis KPC mouse model (*n* = 6 mice, 3 fields assessed per sample). CK19, green; PLXNB3, red; DAPI, blue; scale bars, 50 *μ*m. (b) Real-time PCR measured mRNA levels of SEMA5A and PLXNB3 in human normal pancreas epithelial cell line HPNE, human PDAC cell line PANC1, CAPAN1, BxPC3, and AsPC1 and murine PDAC cell line KPC1199 and Panc02. (c, d) CT combined in vivo imaging with organ reconstruction assessed liver metastasis burdens of intrasplenic injection mouse model (*n* = 6 mice per group). Scale color bars: 5.00 × 10 [4]−3.00 × 10 [5]. Reconstructed liver, red; reconstructed spleen, orange; signals of tumor niches, rainbow. Mean ± s.e.m.; two tailed unpaired *t*-test, ^∗∗^*P* < 0.01. (e, f) Separated KPC1199^shCTRL^ or KPC1199^sh*PLXNB3*^ invaded mouse livers were assessed via in vivo imaging system (*n* = 6 mice per group). Scale color bars: 1.50 × 10 [6]−2.50 × 10 [7]. Rainbow, signals of tumor niches. Mean ± s.e.m.; two tailed unpaired t-test, ^∗∗^*P* < 0.01. (g) IHC-P staining of PLXNB3 (left) or CD163 in liver metastasis of intrasplenic mouse models (*n* = 6 mice, 3 fields assessed per sample). Scale bars, 50 *μ*m. (h) Ki67 and TUNEL staining on liver metastatic niches of intrasplenic injection mouse model were performed (*n* = 6 mice, 3 fields assessed per sample). TUNEL signals, green; Ki67, red; DAPI, blue; scale bars, 50 *μ*m. (i) Prognosis of mouse model bearing KPC1199^shCTRL^ or KPC1199^sh*PLXNB3*^ injection was analyzed via the Kaplan-Meier curve (*n* = 6 mice per group).

We first performed IF staining on KPC mice in which liver metastasis would generate spontaneously at about 4 months old. The metastatic niches of KPC mice performed double positive of CK19 and PLXNB3, indicating that TME of liver metastatic niches of KPC mice was similar to that of patients (Figure 3(a)). Then, we examined SEMA5A and PLXNB3 expressions in PDAC human or murine cell lines (Figure 3(b)). It has been noticed that PLXNB3 were upregulated in most cell lines, indicating the tumor resident behavior of which, while SEMA5A was maintained at a relative low level for its source was usually exogenous. Then, intrasplenic injection model was performed. In this model, the injected tumor cells would begin to generate metastatic niches from left lobe in the liver after passing through the portal vein in accordance with physiological process of PDAC liver metastasis (Figure 3(c)). Metastasis burdens of C57BL/6J mice bearing KPC1199 injection were evaluated via CT combined in vivo imaging with organ reconstruction, and the results of which have demonstrated that RNAi of *PLXNB3* attenuated the outgrowth of liver metastasis (Figures 3(c)–3(f)). Interestingly, the knockdown of *PLXNB3* also reduced the infiltration of CD163-positive M2 TAMs into metastatic niches, illustrating that the promotion effects of tumor cells and TAMs were reciprocal (Figure 3(g)). Further staining also elucidated that PLXNB3 could facilitate cell proliferation and prevent tumor cells from apoptosis (Figure 3(h)). Meanwhile, KM analysis on mice bearing liver metastasis showed that PLXNB3 knockdown significantly prolongs life survival (Figure 3(i)).

Collectively, these data have demonstrated that PLXNB3 facilitated tumor burden growth *in vivo.*

### 3.4. SEMA5A-PLXNB3 Axis Promoted Liver Metastasis *In Vitro*

**Figure 4:**
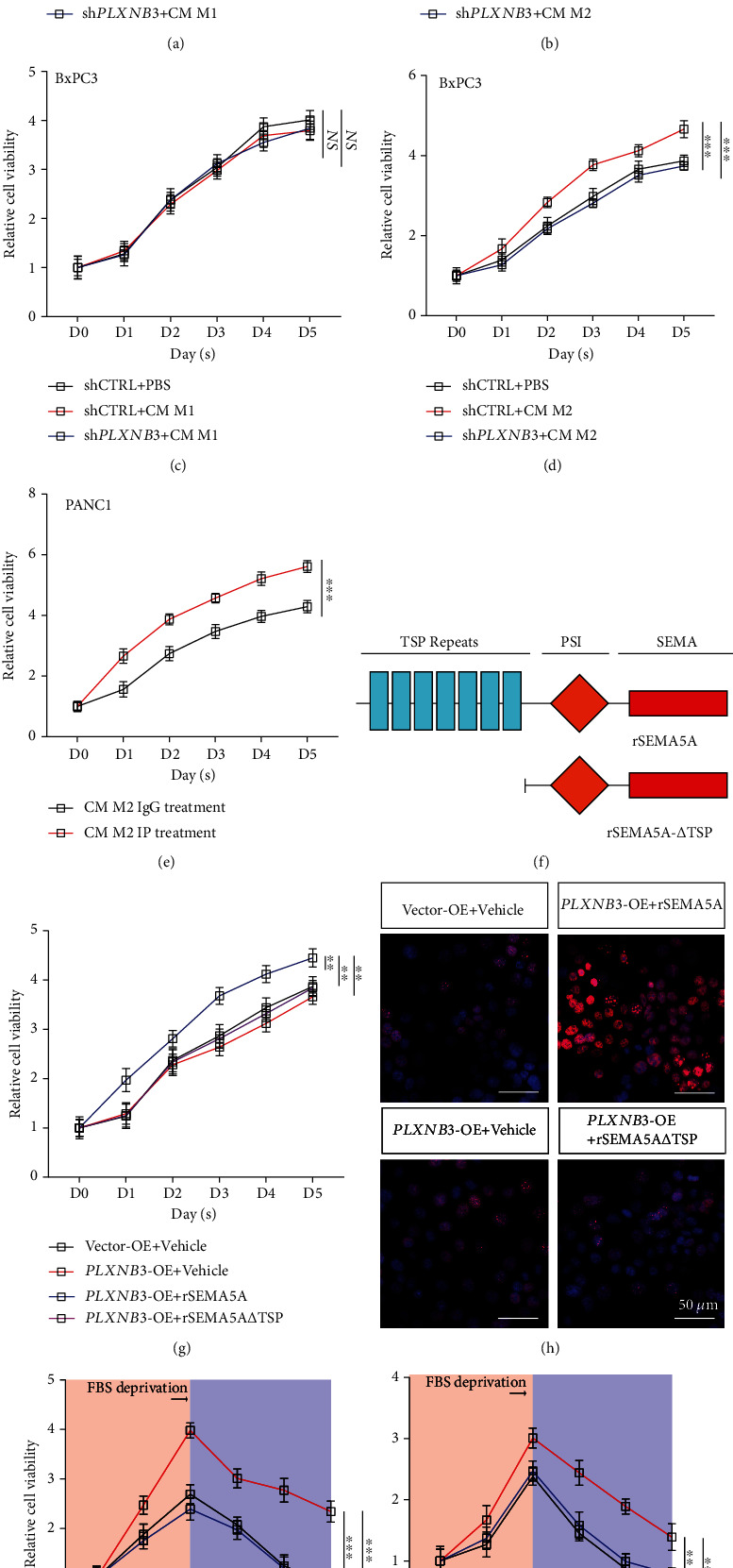
SEMA5A-PLXNB3 axis promotes liver metastasis *in vivo.* (a, b) Relative cell viability of PANC1^shCTRL^ or PANC1^sh*PLXNB3*^ cells cultured with or without M1 TAM (a) or M2 TAM (b) conditional medium measured by CCK-8 kit (*n* = 5 repeats per group, mean ± s.e.m., repeat measures ANOVA). ^∗∗∗^*P* < 0.001; NS: no significance. (c, d) Relative cell viability of BxPC3^shCTRL^ or BxPC3^sh*PLXNB3*^ cells cultured with or without M1 TAM (c) or M2 TAM (d) conditional medium measured by CCK-8 kit (*n* = 5 repeats per group, mean ± s.e.m., repeat measures ANOVA). ^∗∗∗^*P* < 0.001; NS: no significance. (e) Relative cell viability of PANC1 was cultured in M2 TAM conditional medium experienced IP of SEMA5A. (*n* = 5 repeats per group, mean ± s.e.m., repeat measures ANOVA). ^∗∗∗^*P* < 0.001. (f) Structure of SMEA5A full length and SEMA5A-*Δ*TSP. (g) Relative cell viability of AsPC1^*PLXNB3*-OE^ in the presence or absence of SEMA5A and SEMA5A-*Δ*TSP (*n* = 5 repeats per group, mean ± s.e.m., repeat measures ANOVA). ^∗∗^*P* < 0.01. (h) EdU staining was performed on AsPC1^*PLXNB3*-OE^ in the presence or absence of SEMA5A and SEMA5A-*Δ*TSP (3 fields assessed per sample). EdU, red; DAPI, blue; scale bars, 50 *μ*m. (i, j) Cell survival ability was assessed on PANC1 (i) and BxPC3 (j) cell line with administration of rSEMA5A or SEMA5A-*Δ*TSP (*n* = 5 repeats per group, mean ± s.e.m., repeat measures ANOVA). ^∗∗^*P* < 0.01; ^∗∗∗^*P* < 0.001.

Data have pointed out that PANC1 and BxPC3 cell line highly expressed PLXNB3 with SEMA5A deficiency; we next aim to stimulate these two cell lines with conditional medium (CM) of M1 or M2 TAMs. Human THP-1 cell was cultured before phorbol 12-myristate 13-acetate (PMA) treatment followed by polarization via LPS or IFN-*γ* inducement. CCK-8 assays have shown that M1 CM failed to facilitate tumor cell growth as M2 CM did, while the inference of *PLXNB3* could abolish the promotion effect brought by M2 CM (Figures 4(a)–4(d)). Furthermore, the M2 TAM CM would lose its growth stimulation effects on PANC1 tumor cell once SEMA5A was eliminated via immune precipitation (IP) (Figure 4(e)), which have manifested the important roles of SEMA5A in PLXNB3-induced proliferation advantage.

SEMA5A protein contains several domains, and TSP repeats are responsible for the interaction of SEMA5A to PLXNB3 receptor, cutting off which could greatly abate the binding ability of SEMA5A (Figure 4(f)). We next produced recombined SEMA5A (rSEMA5A) and trunked SMEA5A lacking TSP domain (rSEMA5A-*Δ*TSP) for further study [24, 25]. The results of CCK-8 and EdU assays have indicated that the administration of rSEMA5A could significantly enhance the proliferation ability of PLXNB3-overexpressed tumor cell AsPC1^*PLXNB3*-OE^, while this effect would be lost once TSP domain was cut off (Figures 4(g) and 4(h)). What is more, the removement of FBS could induce the apoptosis of tumor cells with vehicle or SEMA5A-*Δ*TSP exposure, while the apoptosis effects were significantly attenuated with treatment of rSEMA5A (Figures 4(i) and 4(j)).

Taken together, these results have indicated that the binding of SEMA5A to PLXNB3 is critical for its proliferation promotive effects.

### 3.5. SEMA5A-PLXNB3 Axis Facilitates Tumor Cell Growth via Enhancing the Warburg Effect

**Figure 5:**
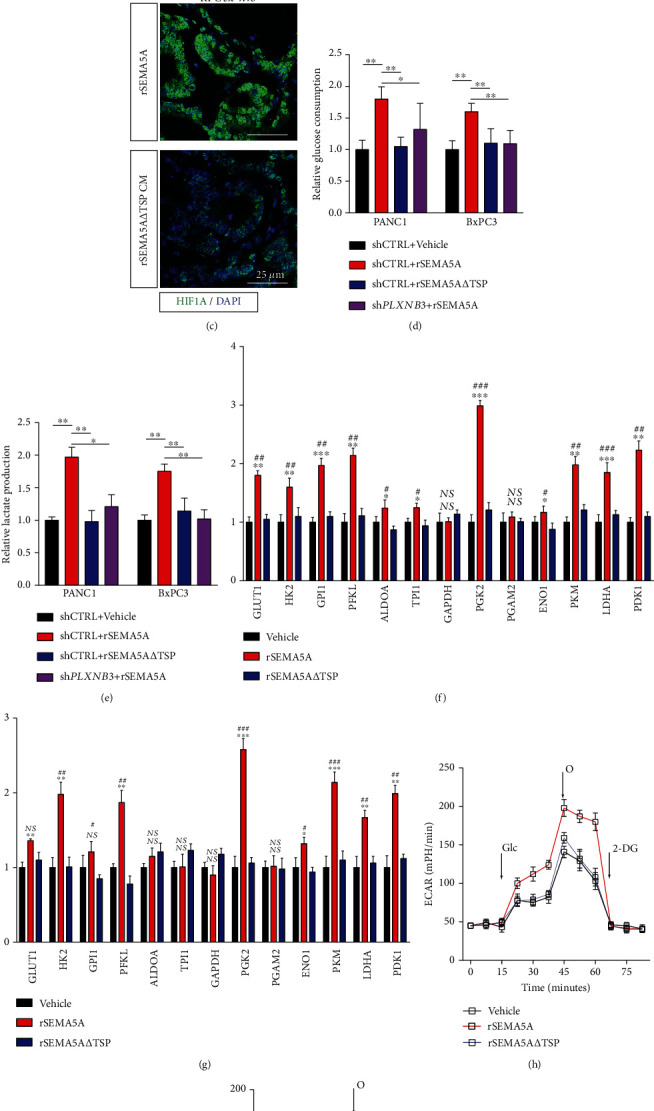
SEMA5A-PLXNB3 axis facilitates the Warburg effects via enhancing glycolysis. (a, b) Relative mRNA level of HIF1A (a) or MYC (b) affected by administration of rSEMA5A or SEMA5A-*Δ*TSP (*n* = 3 repeats per sample; mean ± s.e.m.; two tailed unpaired *t*-test). ^∗∗^*P* < 0.01; ^∗∗∗^*P* < 0.001; NS: no significant difference. (c) IF staining in KPC derived ex vivo liver metastasis tissue treated with rSEMA5A or SEMA5A-*Δ*TSP demonstrating the expression and distributions of HIF-1*α* (*n* = 5 mice per group, 3 fields assessed per sample). HIF1A, green; DAPI, blue; scale bars, 50 *μ*m. (d, e) Metabolism level alterations of PANC1 and BxPC3 cell lines affected by SEMA5A-PLXN3 axis were assessed by relative glucose consumption (d) and lactate production (e) (*n* = 5 repeats per group, mean ± s.e.m.; two tailed unpaired *t*-test). ^∗^*P* < 0.05; ^∗∗^*P* < 0.01; ^∗∗∗^*P* < 0.001. (f, g) The expression alteration of key enzymes involved in glycolysis pathway in PANC1 (f) and BxPC3 (g) after stimulation by rSEMA5A or SEMA5A-*Δ*TSP exposure (*n* = 3 repeats per group, mean ± s.e.m.; two tailed unpaired *t*-test). ^∗^*P* < 0.05, ^∗∗^ *P* < 0.01, and ^∗∗∗^*P* < 0.001 for rSEMA5A group; ^#^*P* < 0.05, ^##^*P* < 0.01, and ^∗∗∗^*P* < 0.001 for SEMA5A-*Δ*TSP group; NS: no significance. (h, i) Relative ECARs in PANC1 (h) or BxPC3 (i) treated with rSEMA5A or SEMA5A-*Δ*TSP measured by seahorse system (*n* = 3 repeats, mean ± s.e.m.).

We next aimed to unveil the mechanism of SEMA5A-PLXNB3 axis mediated cell proliferation effects. We have observed that activated SEMA5A and PLXNB3 axis could enhance the survival ability in medium lacking FBS (Figures 4(i) and 4(j)), and further study has shown that this axis activation could induce the expression of HIF1A and C-MYC which is vital for cell against stress including hypoxia (Figures 5(a) and 5(b)). To validate these results, we performed ex vivo tests. PDAC tumor cell-metastasized livers of KPC mice were sliced in half, one was immersed into complete medium with rSEMA5A, and another one was treated with rSEMA5A-*Δ*TSP. IF staining has displayed that the exposure of rSEMA5A significantly increased the expression of HIF1A and promoted its nuclear location (Figure 5(c)). The Warburg effects are characterized by series of abnormal cell activities, including enhanced glucose consumption, additional lactate production, and aberrant extracellular H^+^ accumulation. We also observed that upregulations of the Warburg effect key enzymes PKM2 or PGK1were coexpressed with KI67 in SEMA5A-enriched tumor niches of human PDAC liver metastasis, demonstrating the enhanced proliferation ability in the Warburg effect area (Supplemental Figure 1A). The results of *ex vivo* on tissues harvested from KPC mice have also confirmed these results (Supplemental Figure 1B).We have demonstrated that treatment of rSEMA5A- on PLXNB3-expressed tumor cell lines upregulated glucose consumption and lactate production (Figures 5(d) and 5(e)). We have also explored the expression alterations of key enzymes involved in glycolysis; the activation of this axis could upregulate the expressions of most enzymes in glycolysis and hypoxia pathway (Figures 5(f) and 5(g)). At last, we consolidated these results in Seahorse system; the results have shown that activated SEMA5A-PLXNB3 axis significantly facilitates the extracellular acid rate (ECAR) value (Figures 5(h) and 5(i)).

Altogether, our results have pushed forward evidence that SEMA5A-PLXNB3 axis promotes tumor cell proliferation via enhancing the Warburg effect.

## 4. Discussion

In our study, we have demonstrated that M2 TAM-derived secreted protein SEMA5A would interact with tumor cell membrane located in PLXNB3 and thus induce tumor cell proliferation for metastatic niche expansion via enhancing the Warburg effects. SEMA family has long been recognized as vital in various kinds of cancers including PDAC, breast cancer, and gastric cancer (GC). Here, we mainly research the roles SEMA5A played in liver metastasis of PDAC.

TAMs are essential components of TME; our results on IHC-P have displayed that numerous CD163^+^ M2 TAMs could be observed staying in ECM of liver metastatic niches. The effects they exert on tumor cells were through secreting SEMA5A. The sources of SEMA family were diverse as follows: CAF derivation, tumor autocrine, and immune cell secretion. Through IHC-P staining, PCR screening, and IF colocation, we then determined that in liver metastasis of PDAC, the source of SEMA5A was M2 TAMs. The relationship analysis also supported our hypothesis. Considering that multiple receptors of SEMA5A could exist in metastatic niches, we also detected potential receptors PLXNA3 and PLXNA4 of SEMA5A [18, 19, 21]. IF tests have demonstrated that only PLXNB3 existed on M2 TAM-infiltrated niches and this receptor could be observed colocation in tumor cells and adjacent ECM. We finally confirmed that it was SEMA5A-PLXNB3 axis activated in M2 TAM-dominated niches.

We performed confirmation on both intrasplenic injection mouse model and spontaneous KPC liver metastasis model to evaluate the function of SEMA5A-PLXNB3 axis. Notably, in liver metastatic niches of KPC mice, the stroma is thicker than that in artificial mouse model; thus, the upregulation of PLXNB3 in metastatic niches is more persuasive. The outgrowth facilitation PLXNB3 exerted on tumor cells was triggered by SEMA5A which was also consolidated by SEMA5A-*Δ*TSP. SEMA5A-*Δ*TSP, which lacked the TSP domain and loses most affinity with PLXNB3, would fail to efficiently bind to PLXNB3 and induce the downstream cell functions, which further provide solid evidence for SEMA5A-PLXNB3 axis.

In mechanism exploration, we have found that this axis could facilitate either the proliferation ability or survival of tumor cells, especially in cell stress. It has been reported that the tumor cells always suffer from fierce struggles for survival due to deficient nutrition, high cell density, and relative low pH value in TME [26–29]. The situation will be even worse in metastatic TME, since alien microenvironments for tumors are harsh for their implantation and outgrowth. Tumor cell will utilize glucose mainly by aerobic glycolysis rather than oxidative phosphorylation; even the oxygen supplement is sufficient, though the latter is much more energetically efficient on generating adenosine triphosphate (ATP). This phenomenon is termed as the Warburg effect [29–32]. Tumor cells choose the Warburg effect not only to meet their needs of rapid energy supplement for exuberant proliferation and growth but also for gaining enough glycolytic intermediates into various biosynthetic pathways to assemble newly birthed cells [33, 34]. Another hypothesis has also unveiled that large amounts of H^+^ were generated as by products and released into TME, causing microenvironment remodeling which could be more adaptive for tumor cells. Thus, enhanced glucose uptake, high concentration of extracellular lactate, and aberrantly upregulated glycolysis enzyme are obvious hallmarks of the Warburg effect. We aimed to check the relationship of events including the Warburg effect, tumor growth, and SEMA5A overexpression. In our liver metastasis samples from PDAC patients, we have observed that it was SEMA5A enriched niche in which high expression of the Warburg effect key enzyme PKM2 or PGK1 existed. Furthermore, the upregulated enzymes colocated with KI67, the proliferation marker, illustrating that the Warburg effect could facilitate tumor growth. We also validated the results in *ex vivo* tests using spontaneous liver metastatic niches from KPC mouse models. SEMA5A, but not the truncated protein rSEMA5A-*Δ*TSP, could induce PKM2 or PGK1 expressions in tumor cells residing in metastatic niches, elucidating the importance of SEMA5A-PLXNB3 axis in the Warburg effect triggering. It was also observed that KI67 staining was much stronger in the rSEMA5A group. These results have unveiled that the activation of SEMA5A-PLXNB3 axis could lead to the Warburg effect which facilitated tumor growth. In our study, we have manifested that the activation of SEMA5A-PLXNB3 axis significantly increased the consumption of glucose and lactate production of PDAC cell lines and stimulated the expression of key enzymes involved in glycolysis pathway. The binding of SEMA5A to PLXNB3 also upregulated ECAR value and further confirmed our hypothesis.

Our study provided evidence that SEMA5A-PLXNB3 axis activation promotes liver metastasis of PDAC via enhancing the Warburg effects, which could be a potential new target for metastasized PDAC therapy.

## Figures and Tables

**Table 1:** Primer sequences used for RT-qPCR.

Gene	Forward primer (5′ to 3′)	Reverse primer (5′ to 3′)
SMEA5A	GAACCGGAAGCGTGTT	CAGTGAGATGTGGGTTGAAG
PLXNB3	GTGCGGAACCTTCAACATTT	AAAGAGCATGGGTGTTGTCC
SEMA3A	GTGCCAAGGCTGAAATTATCCT	CCCACTTGCATTCATCTCTTCT
SEMA3B	ACATTGGTACTGAGTGCATGAAC	GCCATCCTCTATCCTTCCTGG
SEMA3C	TTTGCGTGTTGGTTGGAGTAT	TCCTGTAGTCTAAAGGATGGTGG
SEMA3D	GCAAAGGAACGGGTGGAATTA	TCTGCCAGACTCCAAATTATGTG
SEMA3E	AGGCAGGGACCTTGTATATTCC	TGTACTCGGCCAGTGTATCTC
SEMA3F	AACACAACCGACTACCGAATC	GGCTGCCCAGTGTATAATGAG
SEMA3G	CTCTGCGTTCCGACTCTGAC	CTGACAGTGACATGGTTCGAG
SEMA5B	CCGTGGGTCTCTAACTTCACC	GACTCGCACGTAGTTCTGACA
PLXNA1	ACCCACCTAGTGGTGCATGA	CGGTTAGCGGCATAGTCCA
PLXNA2	CTGAGAATCGTGACTGGACCT	GCTTATAGACCCGGTTGATGG
PLXNA3	AGTCCTGCTATCGTGGGGAG	CAGAAGTTGCCGTTGATCTGC
PLXNA4	GTCATTTGTCACATTCCGAGGA	GCTTGTAAATCCGATTGACGGC
PLXNB1	ACCAACTGCATTCACTCCCAA	GCACTCATCAGGCATCACAG
PLXNB2	AGCCTCTTCAAGGGCATCTG	GCCACGAAAGACTTCTCCCC
GLUT1	ATTGGCTCCGGTATCGTCAAC	GCTCAGATAGGACATCCAGGGTA
HK2	TGATCGCCTGCTTATTCACGG	AACCGCCTAGAAATCTCCAGA
LDHA	GCTCCCCAGAACAAGATTACAG	TCGCCCTTGAGTTTGTCTTC
PKM	TCGCATGCAGCACCTGATT	CCTCGAATAGCTGCAAGTGGTA
GPI1	CAAGGACCGCTTCAACCACTT	CCAGGATGGGTGTGTTTGACC
PFKL	GGTGCCAAAGTCTTCCTCAT	GATGATGTTGGAGACGCTCA
ALDOA	AACTTTCCTCTGCCTAGCCC	GTACAGGCACAGTCGCAGAG
TPI1	AGCTCATCGGCACTCTGAAC	CCACAGCAATCTTGGGATCT
GAPDH	CTGGGCTACACTGAGCACC	AAGTGGTCGTTGAGGGCAATG
PGK2	AAACTGGATGTTAGAGGGAAGCG	GGCCGACCTAGATGACTCATAAG
PGAM2	AGAAGCACCCCTACTACAACTC	TCTGGGGAACAATCTCCTCGT
ENO1	GCCGTGAACGAGAAGTCCTG	ACGCCTGAAGAGACTCGGT

## Data Availability

The datasets used and/or analyzed during the current study are available from the corresponding authors on reasonable request.

## Supplementary Materials

Supplementary Materials Supplemental figure 1: IF staining demonstrating the locations of PKM2/PGK1 and Ki67 expressions in tissues from patients or KPC mouse models.
