# Supplementary material for: SEMA5A-PLXNB3 Axis Promotes PDAC Liver Metastasis Outgrowth through Enhancing the Warburg Effect
Source: J Immunol Res. 2023 Jan 27;2023:3274467. doi: 10.1155/2023/3274467 (PMC9897926; doi:10.1155/2023/3274467)
Supplement: Supplementary Materials — Supplemental figure 1: IF staining demonstrating the locations of PKM2/PGK1 and Ki67 expressions in tissues from patients or KPC mouse models. [file 3274467.f1.docx]

**Supplemental Figure 1. Warburg Effect is observed in SEMA5A enriched tissues and closely related to tumor growth.**

(**A**) The co-expressions of KI67 and Warburg effect related enzymes PKM2 or PGK1 were assessed by IF staining in liver metastatic niches of PDAC patients with different SEMA5A expressions. PKM2 or PGK1, green; KI67, red; DAPI, blue; Scale bars, 50μm. (**B**) IF staining in KPC derived *ex vivo* liver metastasis tissue treated with rSEMA5A or SEMA5A-*Δ*TSP demonstrating the co-expressions of KI67 and Warburg effect related enzymes PKM2 or PGK1. (n=5 mice per group, 3 fields assessed per sample). PKM2 or PGK1, green; KI67, red; DAPI, blue; Scale bars, 50μm.


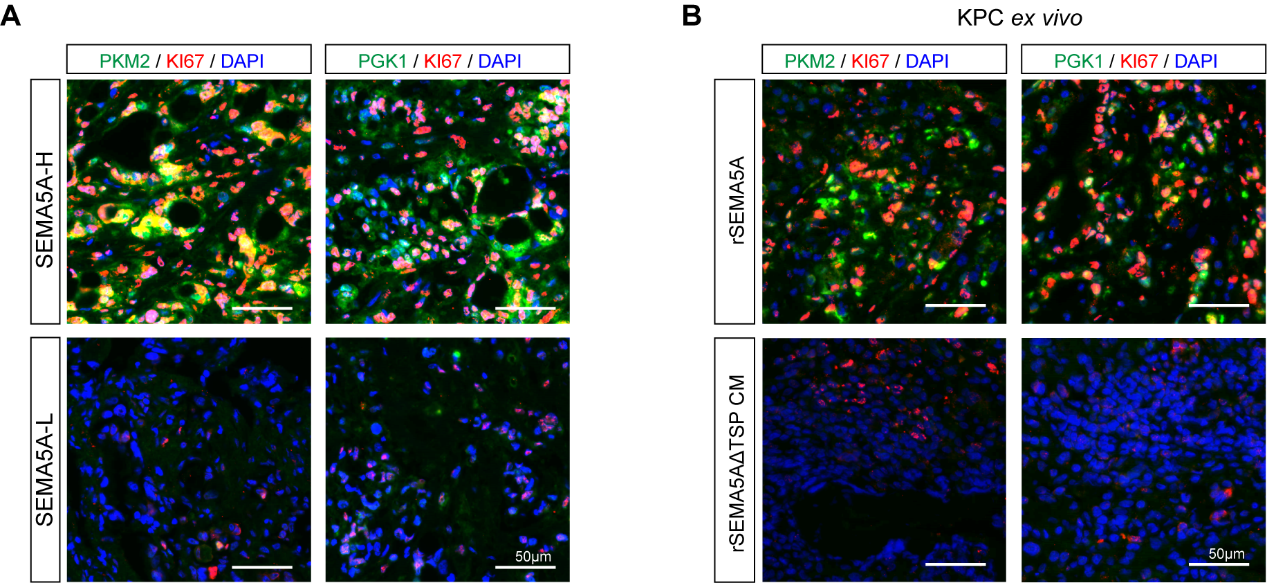
SUPPLEMENTAL FIG1
